# Rurality and Area Deprivation and Outcomes After Out-of-Hospital Cardiac Arrest

**DOI:** 10.1001/jamanetworkopen.2025.3435

**Published:** 2025-04-15

**Authors:** Lakota Cheek, Robert H. Schmicker, Remle Crowe, Emily Goren, Amanda West, Jason McMullan, Colin Raelson, Jeanne Poole, Karen Adams, Antje Hoering, Brent Myers, Graham Nichol

**Affiliations:** 1 Department of Emergency Medicine, University of Washington, Seattle; 2 Department of Biostatistics, University of Washington, Seattle; 3 ESO Inc, Austin, Texas; 4 Cancer Research and Biostatistics, Seattle, Washington; 5 Department of Emergency Medicine, University of Cincinnati, Cincinnati, Ohio; 6 Swedish Heart and Vascular Institute, Swedish Cherry Hill Campus, Seattle, Washington; 7 Department of Medicine, University of Washington, Seattle; 8 University of Washington–Harborview Center for Prehospital Emergency Care, Seattle

## Abstract

**Importance:**

Large regional variations in outcomes after out-of-hospital cardiac arrest (OHCA) exist.

**Objective:**

To assess whether neighborhood rurality or economic deprivation where an OHCA occurred is associated with variation in emergency medical services (EMS) outcomes after OHCA.

**Design, Setting, and Participants:**

This cohort study used data collated by ESO Inc on US adult patients (aged ≥18 years) with nontraumatic OHCA receiving chest compressions or defibrillation from EMS between January 1, 2022, and December 31, 2023.

**Exposures:**

Rurality was assessed using Rural-Urban Commuting Area codes. Deprivation was assessed using the Area Deprivation Index. Both were derived from US Census data and grouped by EMS agency.

**Main Outcomes and Measures:**

Outcomes were restoration of spontaneous circulation (ROSC) at emergency department (ED) arrival, survival to hospital discharge, and favorable discharge destination. Discharge outcomes were only available for patients transported to hospitals using health data exchange. Generalized estimating equations were used to account for correlated data.

**Results:**

A total of 162 289 patients with OHCA had resuscitation attempted (median [IQR] age, 66 [53-76] years; 62.3% male). Overall, 28.1% of these patients lived in rural or suburban locations, 12.3% lived in areas with high deprivation, 18.7% had a first rhythm of ventricular tachycardia or ventricular fibrillation or shockable by automated external defibrillator rhythm, and 27.6% received bystander cardiopulmonary resuscitation. The mean (SD) EMS response time was 8.7 (5.6) minutes. Upon arrival at the ED, 23.7% of patients had ROSC. Compared with OHCAs in urban areas with low deprivation, those in rural areas with high deprivation (adjusted odds ratio [AOR], 0.81; 95% CI, 0.72-0.91), moderate deprivation (AOR, 0.75; 95% CI, 0.70-0.81), or low deprivation (AOR, 0.74; 95% CI, 0.62-0.88) had lower odds of ROSC at ED arrival. Among patients transported to hospitals using health data exchange, OHCAs in urban areas with high or moderate deprivation had lower odds of survival (AOR, 0.78 [95% CI, 0.68-0.90] and 0.82 [95% CI, 0.75-0.89], respectively) and favorable discharge destination (AOR, 0.65 [95% CI, 0.53-0.79] and 0.77 [95% CI, 0.69-0.87], respectively).

**Conclusions and Relevance:**

In this cohort study, OHCAs in rural areas of all levels of economic deprivation were associated with less ROSC at ED arrival vs urban areas with low deprivation, and OHCAs in urban areas with high or moderate deprivation are associated with less survival and less favorable discharge destination, suggesting worse neurologic outcomes. Care improvements alone may not reduce geographic differences in outcomes after OHCA.

## Key Points

**Question:**

What is the association of neighborhood rurality or economic deprivation with regional variation in outcomes after out-of-hospital cardiac arrest (OHCA)?

**Findings:**

In this cohort study of 162 289 adult patients, those with OHCA in rural areas had lower odds of achieving restoration of spontaneous circulation at emergency department arrival vs urban areas with a low deprivation index. Urban areas with a high deprivation index had lower survival rates and less favorable discharge destinations, suggesting worse neurologic outcomes.

**Meaning:**

These findings suggest that improvements in care delivery alone may not eliminate geographic differences in OHCA outcomes.

## Teaser

This cohort study assesses whether neighborhood rurality and economic deprivation are associated with outcomes up to hospital discharge among patients treated by emergency medical services after out-of-hospital cardiac arrest.

## Introduction

There are large regional variations in outcomes after out-of-hospital cardiac arrest (OHCA) across communities.^1,2^ These outcomes are not fully explained by differences in patient characteristics or emergency medical services (EMS) treatment received.^3^ Differences in outcome after OHCA were previously associated with differences in social determinants of health.^4,5,6,7,8^ Experts have identified that disparities in health outcomes are increasing in the US^9^ and that efforts are needed to reduce them.^10^

Approximately 14% to 20% of Americans live in a rural area.^11,12^ Rural populations have worse health outcomes than urban populations.^13,14^ Poverty is independently associated with increased all-cause mortality.^15^ Outcomes differ among patients with cardiovascular disease according to the rurality or economic deprivation of their neighborhood of residence.^16,17^

To date, there is incomplete knowledge about whether regional variations in outcomes after OHCA are independently associated with the rurality or economic deprivation of the neighborhood in which the arrest occurred. If such an association exists, strategic allocation of clinical and nonclinical resources might reduce geographic disparities in outcomes after OHCA. We assessed whether rurality and economic deprivation explain regional variation in outcomes of care for patients with OHCA.

## Methods

### Study Design, Setting, and Population

This cohort study included adults aged 18 years or older with nontraumatic OHCA and chest compressions or defibrillation by EMS from January 1, 2022, to December 31, 2023. These dates were based on when health data exchange was initiated within the ESO Inc software used. The St David’s Healthcare Institutional Review Board determined that collation of these data were exempt from human participants research regulations, as did the University of Washington Institutional Review Board for the data analysis. Participants did not give informed consent for enrollment in this quality improvement registry. This study followed the Strengthening the Reporting of Observational Studies in Epidemiology (STROBE) reporting guideline.

The study included EMS agencies that use electronic medical record software developed by ESO Inc. Adult patients with OHCA were defined as those assessed by organized EMS personnel, did not have a documented traumatic injury, and received attempts of external defibrillation or chest compressions by EMS personnel. Patients for whom care was transferred to a nonparticipating EMS agency were excluded as lost to follow-up.

### Data Source

The Informatics to Improve Emergency Resuscitation Registry–OHCA uses contemporary data collated in real time to characterize patient characteristics (eg, sex) and the process (eg, EMS response time) and outcomes of EMS care of patients with OHCA up to transfer of care to a receiving hospital’s emergency department (ED).^18^ As of December 1, 2023, this electronic medical record software is used by more than 2500 agencies that respond to more than 30% of all 911 EMS transports in the US, including all 50 states and the District of Columbia. It includes health data exchange with more than 1000 receiving hospitals to allow the encounter to be described up to hospital discharge. No manual data entry is required beyond routine documentation of care. This electronic medical record has built-in range and logic checks to improve data quality. The Informatics to Improve Emergency Resuscitation Registry–OHCA extends the ESO Data Collaborative, which is adaptable for quality improvement and research purposes.

### Rurality, Economic Deprivation, and Covariates

Rurality was assessed using Rural-Urban Commuting Area (RUCA) codes,^19^ which classify census tracts using measures of population density, urbanization, and daily commuting. The most recent RUCA codes are based on data from the 2010 decennial census and the 2006-2010 American Community Surveys. The RUCA codes are categorized using whole numbers (1-10) to describe metropolitan, micropolitan, small town, and rural commuting areas. Urban, suburban, and rural tracts are defined by RUCA codes 1, 2 to 6, and 7 to 10, respectively, and were grouped at the EMS agency level.

Economic deprivation was assessed using the Area Deprivation Index (ADI),^20,21,22^ which classifies the resources, income, education, and housing status of a geographic area using 17 variables from 5-year American Community Survey estimates.^23,24,25^ The ADI score is continuous and scaled by 10, with higher scores indicating greater deprivation. Low, moderate, and high ADI scores were defined a priori as 50th percentile or less, 50th to 90th percentile, and greater than 90th percentile, respectively, based on previously used cut points.^26^ For our analysis, ADI scores were grouped at the EMS agency level.

All variables were predefined using definitions from the National Emergency Medical Services Information System data dictionary.^27^ Patient race and ethnicity were based on EMS personnel impression and were grouped as Asian, Black, Hispanic or Latino, White, and other (including American Indian or Alaska Native and Native Hawaiian or Pacific Islander).

### Outcomes

The primary outcome was restoration of spontaneous circulation at ED arrival, which was assessed as spontaneous pulse and blood pressure. Survival to discharge was assessed as alive at discharge from the hospital to home, a nursing facility, or a rehabilitation facility. Patients transferred to another acute care facility (eg, to undergo implantable defibrillator placement) were considered to have been discharged alive.

Favorable discharge destination was assessed as discharge home or to a facility with custodial or supportive care or to the care of an organized home health service as a proxy for favorable neurologic outcome. We inferred discharge destination as a proxy for neurologic outcome as follows: discharged to home (Cerebral Performance Category [CPC] 1), discharged to a facility with custodial or supportive care or under care of an organized home health service (CPC 2), discharged to skilled nursing facility (CPC 3), ongoing hospitalization or discharged to hospice (CPC 4), or deceased (CPC 5).

### Statistical Analysis

The primary analyses included all eligible patients with available outcome data. Since some hospitals do not routinely share hospital data with ESO Inc, secondary analyses of post–ED arrival outcomes were restricted to patients transported to hospitals that used the health data exchange. Analyses used urban areas with low deprivation as the reference group because our a priori hypothesis was that these areas would have the best outcomes.

Analyses assessed whether rurality or area deprivation were associated with outcome by using generalized estimating equations^28^ to estimate population-level inferences within EMS agencies because the health of residents (eg, outcome after OHCA) in the same neighborhood may be correlated, thus violating independence assumptions made by regression procedures. Sensitivity analysis used mixed models^29^ to estimate individual-level outcomes.

The primary analysis considered rurality and area deprivation, classified with 8 dummy variables. The analysis plan was defined a priori. A term describing the interaction between rurality and deprivation was included in all models because of the anticipated synergistic association between the 2 factors. Analyses adjusted for measured, potential confounders, including patient and EMS treatment characteristics, in addition to measures of rurality or area deprivation under consideration. These confounders included age, sex, race and ethnicity, witnessed status, location of arrest, bystander cardiopulmonary resuscitation status, first recorded rhythm (ventricular tachycardia or ventricular fibrillation or shockable vs nonshockable by an automated external defibrillator), EMS response to arrival-on-scene time interval, and EMS transport time from scene to receiving hospital (only for patients transported to the hospital).

All analyses were performed using R, version 4.1.1 (R Foundation). Standard methods were used to account for missing data,^30,31^ perform regression diagnostics,^31,32,33,34^ and assess goodness of fit.^31,35,36,37^ For discrimination, a C statistic less than 0.7 was defined a priori as not useful, values higher than 0.7 were considered fair, and values higher than 0.9 were considered excellent.^38^ *P* ≤ .05 was considered statistically significant.

## Results

**Figure 1.**
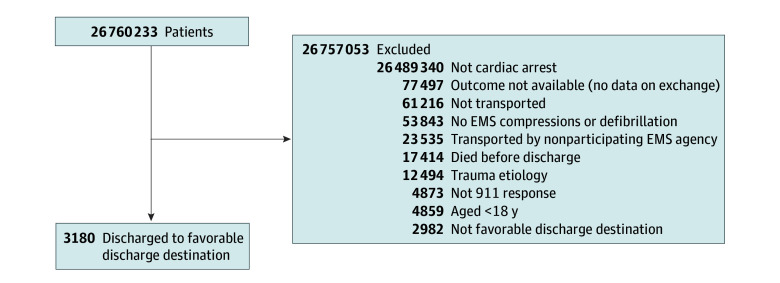
Patient Flow Diagram EMS indicates emergency medical services.

**Figure 2.**
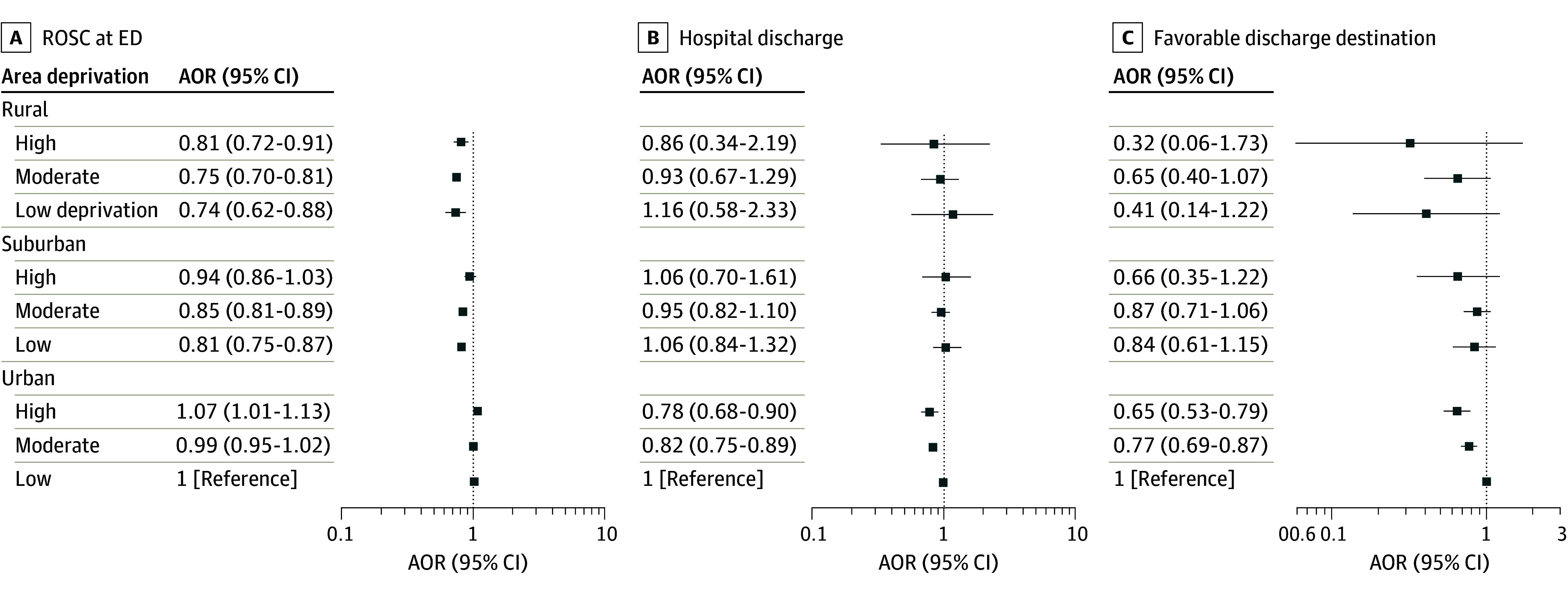
Associations Between Rurality or Deprivation and Outcomes AOR indicates adjusted odds ratio; ED, emergency department; ROSC, restoration of spontaneous circulation.

A total of 162 289 adults with nontraumatic OHCA were included (median [IQR] age, 66 [53-76] years; 37.6% female, 62.2% male, and 0.2% unknown sex; 1.9% reported as Asian, 20.9% as Black, 7.6% as Hispanic or Latino, 69.0% as White, and 10.5% as other or unknown race and ethnicity) (Figure 1; Table 1). Overall, 28.1% of these patients lived in rural or suburban areas, 12.3% lived in areas with high deprivation, 18.7% had a first rhythm of ventricular tachycardia or ventricular fibrillation or shockable by an automated external defibrillator, 38.7% were witnessed by bystanders, and 27.6% received bystander cardiopulmonary resuscitation. The mean (SD) EMS response time was 8.7 (5.6) minutes. Figure 2 shows a graphical representation of the associations between rurality or deprivation and outcomes.

**Table 1.** Patient and EMS Characteristics

Characteristic	Patient, No. (%)			
	Overall (N = 162 289)	Rural (n = 11 337)	Suburban (n = 34 191)	Urban (n = 116 619)
Area deprivation^a^				
High (ADI >90th percentile)	19 067 (12.3)	2212 (21.2)	3888 (12.0)	12 944 (11.6)
Moderate (ADI 50th-90th percentile)	75 378 (48.7)	7008 (67.1)	21 247 (65.4)	47 107 (42.1)
Low (ADI <50th percentile)	60 469 (39.0)	1228 (11.8)	7341 (22.6)	51 872 (46.3)
Age group, y				
18-39	17 614 (10.9)	951 (8.4)	3273 (9.6)	13 382 (11.5)
40-64	58 690 (36.2)	4118 (36.3)	12 822 (37.5)	41 689 (35.7)
≥65	85 985 (53.0)	6268 (55.3)	18 096 (52.9)	61 548 (52.8)
Age, median (IQR), y	66 (53-76)	66 (56-76)	66 (54-75)	66 (52-77)
Sex^b^				
Female	61 024 (37.6)	4022 (35.5)	12 740 (37.3)	44 215 (38.0)
Male	100 994 (62.2)	7303 (64.5)	21 402 (62.7)	72 195 (62.0)
Unknown	265 (0.2)	12 (0.1)	48 (0.1)	204 (0.2)
Race and ethnicity, No. (%)				
Asian, non-Hispanic	2725 (1.9)	14 (0.1)	156 (0.5)	2545 (2.4)
Black, non-Hispanic	30 526 (20.9)	1172 (11.1)	3976 (12.7)	25 348 (24.3)
Hispanic or Latino	11 159 (7.6)	443 (4.2)	1572 (5.0)	9131 (8.8)
White, non-Hispanic	100 952 (69.0)	8834 (83.6)	25 582 (81.4)	66 459 (63.8)
Other race, non-Hispanic^c^	963 (0.7)	109 (1.0)	138 (0.4)	715 (0.7)
Unknown	15 964 (9.8)	765 (6.7)	2767 (8.1)	12 421 (10.7)
Location, No. (%)				
Public	18 191 (11.4)	1254 (11.4)	3313 (9.9)	13 557 (11.8)
Private	140 929 (88.6)	9755 (88.6)	30 024 (90.1)	101 080 (88.2)
Unknown	3169 (2.0)	328 (2.9)	854 (2.5)	1982 (1.7)
Initial rhythm				
VT or VF or shockable by AED	30 347 (18.7)	2258 (19.9)	6834 (20.0)	21 224 (18.2)
Asystole	88 189 (54.3)	6044 (53.3)	18 340 (53.6)	63 733 (54.7)
PEA	33 554 (20.7)	2087 (18.4)	6841 (20.0)	24 593 (21.1)
AED nonshockable	6417 (4.0)	637 (5.6)	1266 (3.7)	4508 (3.9)
Could not determine	3782 (2.3)	311 (2.7)	910 (2.7)	2561 (2.2)
Witness status				
EMS	23 871 (14.8)	1843 (16.3)	5503 (16.1)	16 507 (14.2)
Bystander	62 541 (38.7)	5060 (44.8)	14 660 (43.0)	42 758 (36.8)
Not witnessed	75 311 (46.6)	4397 (38.9)	13 926 (40.9)	56 932 (49.0)
Bystander CPR	44 588 (27.6)	3710 (32.8)	10 786 (31.6)	30 049 (25.9)
AED by layperson	6947 (5.6)	601 (7.3)	1451 (5.9)	4861 (5.3)
Call to EMS arrival on scene, mean (SD), min	8.7 (5.6)	10.8 (7.5)	10.4 (6.6)	8.0 (4.9)
Etiology of arrest				
Presumed cardiac	125 291 (77.4)	8770 (77.5)	26 134 (76.6)	90 274 (77.6)
Noncardiac	36 667 (22.6)	2545 (22.5)	7975 (23.4)	26 118 (22.4)
Unknown	331 (0.2)	22 (0.2)	82 (0.2)	227 (0.2)
Initial service level				
BLS	10 104 (6.4)	845 (7.5)	1673 (5.0)	7577 (6.7)
ALS	145 030 (92.1)	10 026 (89.2)	30 935 (91.9)	103 940 (92.4)

Abbreviations: AED, automated external defibrillator; ALS, advanced life support; BLS, basic life support; CPR, cardiopulmonary resuscitation; EMS, emergency medical services; PEA, pulseless electrical activity; VF, ventricular fibrillation; VT, ventricular tachycardia.

^a^ A total of 7375 had missing data for deprivation, and 142 patients had missing data for rurality.

^b^ Counts and percentages for male and female are out of the total with known sex (n = 162 024), whereas the counts and percentages for unknown sex are out of the total population (N = 162 289).

^c^ Includes American Indian or Alaska Native and Native Hawaiian or Pacific Islander.

### ED Restoration of Spontaneous Circulation

Upon ED arrival, 37 822 of 159 645 patients (23.7%) had restoration of spontaneous circulation (ROSC) (Table 2). Compared with OHCA in urban areas with low deprivation, rural areas with high deprivation (adjusted odds ratio [AOR], 0.81; 95% CI, 0.72-0.91), moderate deprivation (AOR, 0.75; 95% CI, 0.70-0.81), or low deprivation (AOR, 0.74; 95% CI, 0.62-0.88) had lower odds of ROSC at ED arrival (Table 3). Compared with the same reference group, OHCA in suburban areas with high deprivation were not associated with ROSC at ED arrival (AOR, 0.94; 95% CI, 0.86-1.03), but suburban areas of moderate deprivation (AOR, 0.85; 95% CI, 0.82-0.89) or low deprivation (AOR, 0.81; 95% CI, 0.75-0.87) were associated with ROSC at ED arrival. For OHCA in urban areas with high deprivation (AOR, 1.07; 95% CI, 1.01-1.13) or moderate deprivation (AOR, 0.99; 95% CI, 0.95-1.02) no association was found for ROSC at ED arrival. The adjusted model had a C statistic of 0.69, and *P* = .55 for the interaction between rurality and deprivation.

**Table 2.** Patient Outcomes by Rurality and Area Deprivation^a^

Outcome	Patients, No. (%)						
	Overall (N = 162 289)	Rural (n = 11 337)	Suburban (n = 34 191)	Urban (116 619)	High deprivation (n = 19 067)	Moderate deprivation (n = 75 378)	Low deprivation (n = 60 469)
ROSC at ED arrival	37 822 (23.7)	2362 (21.3)	7654 (22.8)	27 768 (24.2)	4457 (23.7)	17 167 (23.2)	14 265 (24.0)
Transported to ED	101 073 (62.3)	7527 (66.4)	22 214 (65.0)	71 229 (61.1)	12 894 (67.6)	48 781 (64.7)	34 519 (57.1)
Transported to ED with health data exchange	23 576 (14.5)	392 (3.5)	2660 (7.8)	20 510 (17.6)	2586 (13.6)	10 298 (13.7)	9502 (15.7)
EMS time from scene to ED arrival, mean (SD), min^b^	10.8 (8.0)	14.4 (2.3)	13.8 (10.4)	9.5 (5.9)	9.5 (7.8)	11.1 (8.5)	10.8 (7.0)
Survival to hospital discharge^b^	6162 (26.1)	123 (31.4)	807 (30.3)	5228 (25.5)	559 (21.6)	2491 (24.2)	2738 (28.8)
Discharge disposition^b^							
Favorable discharge destination							
Any	3180 (14.2)	48 (13.6)	418 (17.1)	2713 (13.8)	247 (10.0)	1250 (12.8)	1490 (16.4)
Home	2814 (12.6)	46 (13.0)	387 (15.9)	2380 (12.1)	222 (9.0)	1120 (11.5)	1297 (14.3)
Custodial or supportive care	366 (1.6)	2 (0.6)	31 (1.3)	333 (1.7)	25 (1.0)	130 (1.3)	193 (2.1)
Skilled nursing facility	821 (3.7)	15 (4.2)	64 (2.6)	742 (3.8)	87 (3.5)	318 (3.3)	385 (4.2)
Ongoing hospitalization or hospice	1006 (4.5)	22 (6.2)	103 (4.2)	878 (4.5)	119 (4.8)	409 (4.2)	435 (4.8)
Deceased	17 608 (78.5)	273 (77.1)	1880 (77.1)	15 445 (78.7)	2040 (82.3)	7861 (80.4)	6856 (75.5)

Abbreviations: ED, emergency department; EMS, emergency medical services; ROSC, restoration of spontaneous circulation.

^a^ As measured using the Area Deprivation Index for which low, moderate, and high scores were defined a priori as 50th percentile or less, 50th to 90th percentile, and greater than 90th percentile, respectively.

^b^ Patients who were transported to the ED and had an available outcome at hospital discharge as hospital used health data exchange with ESO Inc.

**Table 3.** Area Deprivation and Rurality vs Outcome, Adjusted for Covariates Using Generalized Estimating Equations

Outcome	Unadjusted OR (95% CI)			Adjusted OR (95% CI)		
	ROSC at ED (n = 128 641)	Survival to discharge (n = 18 125)	Favorable discharge destination (n = 17 347)	ROSC at ED (n = 128 641)	Survival to discharge (n = 18 125)	Favorable discharge destination (n = 17 347)
**Rurality**						
Rural						
Area deprivation^a^						
High	0.86 (0.77-0.97)	0.75 (0.28-1.98)	0.30 (0.04-2.25)	0.81 (0.72-0.91)	0.86 (0.34-2.19)	0.32 (0.06-1.73)
Moderate	0.79 (0.74-0.85)	1.02 (0.75-1.41)	0.85 (0.54-1.35)	0.75 (0.70-0.81)	0.93 (0.67-1.29)	0.65 (0.40-1.07)
Low	0.79 (0.67-0.94)	1.42 (0.77-2.63)	0.70 (0.25-1.96)	0.74 (0.62-0.88)	1.16 (0.58-2.33)	0.41 (0.14-1.22)
Suburban						
Area deprivation^a^						
High	0.98 (0.91-1.07)	1.16 (0.79-1.70)	0.86 (0.49-1.50)	0.94 (0.86-1.03)	1.06 (0.70-1.61)	0.66 (0.35-1.22)
Moderate	0.90 (0.86-0.94)	1.02 (0.90-1.17)	1.07 (0.90-1.28)	0.85 (0.81-0.89)	0.95 (0.82-1.10)	0.87 (0.71-1.06)
Low	0.86 (0.80-0.92)	1.09 (0.89-1.33)	0.97 (0.73-1.28)	0.81 (0.75-0.87)	1.06 (0.84-1.32)	0.84 (0.61-1.15)
Urban						
Area deprivation^a^						
High	1.01 (0.96-1.06)	0.73 (0.64-0.82)	0.60 (0.50-0.71)	1.07 (1.01-1.13)	0.78 (0.68-0.90)	0.65 (0.53-0.79)
Moderate	0.97 (0.94-1.00)	0.77 (0.71-0.84)	0.72 (0.65-0.80)	0.99 (0.95-1.02)	0.82 (0.75-0.89)	0.77 (0.69-0.87)
Low	1 [Reference]	1 [Reference]	1 [Reference]	1 [Reference]	1 [Reference]	1 [Reference]
**Patient characteristics**						
Aged ≥65 y	NA	NA	NA	0.84 (0.81-0.86)	0.63 (0.59-0.68)	0.40 (0.36-0.45)
Sex						
Female	NA	NA	NA	1 [Reference]	1 [Reference]	1 [Reference]
Male	NA	NA	NA	0.77 (0.74-0.79)	0.87 (0.80-0.94)	0.99 (0.89-1.11)
Race						
Asian, non-Hispanic	NA	NA	NA	1.18 (1.08-1.31)	1.12 (0.87-1.44)	1.00 (0.71-1.41)
Black, non-Hispanic	NA	NA	NA	0.85 (0.82-0.88)	0.94 (0.86-1.04)	0.89 (0.78-1.02)
Hispanic	NA	NA	NA	0.95 (0.90-1.00)	1.09 (0.95-1.24)	1.07 (0.89-1.28)
White, non-Hispanic	NA	NA	NA	1 [Reference]	1 [Reference]	1 [Reference]
Other race, non-Hispanic	NA	NA	NA	0.80 (0.66-0.95)	0.86 (0.56-1.32)	0.86 (0.46-1.61)
Location						
Public	NA	NA	NA	1.30 (1.24-1.35)	1.35 (1.23-1.48)	1.60 (1.41-1.81)
Private	NA	NA	NA	1 [Reference]	1 [Reference]	1 [Reference]
Bystander CPR	NA	NA	NA	1.05 (1.02-1.09)	1.09 (1.00-1.19)	1.19 (1.05-1.34)
No	NA	NA	NA	1 [Reference]	1 [Reference]	1 [Reference]
Yes	NA	NA	NA	2.02 (1.95-2.08)	1.65 (1.50-1.82)	1.96 (1.70-2.27)
EMS	NA	NA	NA	2.39 (2.29-2.50)	1.99 (1.77-2.23)	2.66 (2.25-3.15)
Initial rhythm						
VT/VF	NA	NA	NA	1 [Reference]	1 [Reference]	1 [Reference]
Asystole	NA	NA	NA	0.40 (0.39-0.41)	0.22 (0.20-0.24)	0.15 (0.13-0.18)
PEA	NA	NA	NA	0.87 (0.84-0.90)	0.46 (0.42-0.51)	0.35 (0.31-0.39)
EMS arrival time	NA	NA	NA	0.99 (0.99-0.99)	0.97 (0.96-0.99)	0.98 (0.96-1.01)
Transport time	NA	NA	NA	NA^b^	1.01 (1.00-1.02)	1.02 (1.01-1.03)
C statistic	NA	NA	NA	0.69	0.72	0.78
*P* value for interaction between rurality and deprivation	NA	NA	NA	0.55	0.88	0.41

Abbreviations: CPR, cardiopulmonary resuscitation; ED, emergency department; EMS, emergency medical services; NA, not applicable; PEA, pulseless electrical activity; ROSC, restoration of spontaneous circulation; VT/VF, ventricular tachycardia/ventricular fibrillation.

^a^ As measured using the Area Deprivation Index for which low, moderate, and high scores were defined a priori as 50th percentile or less, 50th to 90th percentile, and greater than 90th percentile, respectively.

^b^ Transport time not included in model of ROSC at ED, as patients pronounced dead in field were not transported to hospital.

#### Survival to Hospital Discharge

Among patients transported to hospitals using health data exchange, 6162 of 23 576 (26.1%) survived to hospital discharge (Table 2). Compared with OHCA in urban areas with low deprivation, OHCA in rural areas with high deprivation (AOR, 0.86; 95% CI, 0.34-2.19), moderate deprivation (AOR, 0.93; 95% CI, 0.67-1.29), or low deprivation (AOR, 1.16; 95% CI, 0.58-2.33) did not have significantly lower odds of survival (Table 3). Similarly, OHCA in suburban areas with high deprivation (AOR, 1.06; 95% CI, 0.70-1.61), moderate deprivation (AOR, 0.95; 95% CI, 0.82-1.10), or low deprivation (AOR, 1.06; 95% CI, 0.84-1.32) did not have significantly lower odds of survival. Patients with OHCA in urban areas with high deprivation (AOR, 0.78; 95% CI, 0.68-0.90) or moderate deprivation (AOR, 0.82; 95% CI, 0.75-0.89) had lower odds of survival compared with those in urban areas with low deprivation. The adjusted model had a C statistic of 0.72, and *P* = .88 for the interaction between rurality and deprivation.

#### Favorable Discharge Destination

A total of 3180 of 22 615 patients (14.2%) had a favorable discharge destination (Table 2). Compared with patients with OHCA in urban areas with low deprivation, those in rural areas with high deprivation (AOR, 0.32; 95% CI, 0.06-1.73), moderate deprivation (AOR, 0.65; 95% CI, 0.40-1.07), or low deprivation (AOR, 0.41; 95% CI, 0.14-1.22) did not have significantly lower odds of a favorable discharge destination (Table 3). Similarly, OHCA in suburban areas with high deprivation (AOR, 0.66; 95% CI, 0.35-1.22), moderate deprivation (AOR, 0.87; 95% CI, 0.71-1.06), or low deprivation (AOR, 0.84; 95% CI, 0.61-1.15) did not have significantly lower odds of a favorable discharge destination. However, patients with OHCA in urban areas with high deprivation (AOR, 0.65; 95% CI, 0.53-0.79) or moderate deprivation (AOR, 0.77; 95% CI, 0.69-0.87) had significantly lower odds of a favorable discharge destination compared with those in urban areas with low deprivation. The adjusted model had a C statistic of 0.78, and *P* = .41 for the interaction between rurality and deprivation.

### Sensitivity Analyses

The sensitivity analyses were robust when mixed models were used in place of generalized estimating equations (eTables 1-3 in Supplement 1). As well, associations between rurality or deprivation and outcome were similar when each was included separately in models (eFigures 1-6 in Supplement 1).

## Discussion

This cohort study found that OHCAs in rural areas with all levels of economic deprivation were associated with lower odds of ROSC at ED arrival compared with those in urban areas with low deprivation. As well, patients with OHCA in urban areas with moderate or high deprivation had lower odds of survival to discharge and a favorable discharge destination compared with urban areas with low deprivation. The association between rurality or deprivation and outcome was attenuated by these factors’ collinearity, the sparseness of some combinations of the 2 (eg, only 1.4% of OHCAs were in rural areas with high deprivation), the small sample size for assessments of hospital outcomes, and the low incidence of each outcome.

Differences in the incidence, process, and outcome of EMS and hospital-based care for OHCA are associated with patient factors,^4,39,40,41^ EMS interventions,^42,43^ neighborhood rurality,^44,45^ or socioeconomic status.^5,46,47^ These prior observations in multiple geographically separate locations imply that our observations are robust and require a concerted effort to modify. Our study extends this previous work by showing that rurality and neighborhood deprivation are both independently associated with outcomes after OHCA. Other studies recently described multiple changes in EMS response that could be implemented to improve outcomes after OHCA.^48^ However, the association of rurality and deprivation with outcome suggests that changes in EMS response alone may be insufficient to reduce the large and important disparities in outcomes that exist for what continues to be a common public health problem. Nonclinical interventions may be necessary to address the association of rurality and poverty with poor nutrition, poor living conditions, stress, and limited health care access. A key next step should be to assess whether strategic allocation of clinical and nonclinical resources improve overall outcome and reduce health disparities. As well, future work should assess whether these associations hold for other health conditions.

An ongoing challenge to clinical quality improvement and research related to EMS care in the US is the absence of a nationally representative, timely, efficient, adaptable framework for describing the continuum of care from initial call to aid through delivery of prehospital emergency care to hospital discharge. The parent registry used for this analysis may represent a path forward to such a framework.

Our analytic methods were defined a priori to increase the reproducibility of our results.^49^ Modifications made to this a priori plan included adding witnessed status as a covariate for all analyses and transport time from scene to ED arrival as a covariate for analyses including patients transported to the hospital. The former change was made because witnessed status was inadvertently left out of the analysis plan. The latter change was made as transport time is a surrogate for proximity of the arrest to the hospital and is associated with outcomes after OHCA.^50^ Note that transport time was only included in analyses of survival to discharge as it was not available for patients who died in the field. Another change was to the graphical representation of the results from a planned heat map to a forest plot, as ESO restricts the unit of analysis to reduce the likelihood of identifying individual participating EMS agencies. A final change was to not report hospital-free survival as an outcome to simplify the presentation.

Both generalized estimating equations and mixed models were previously used in place of traditional regression approaches to evaluate correlated data. Although previous literature has favored the mixed models, generalized estimating equations have been recommended due to their more straightforward interpretation and their need for fewer assumptions.^51^ Thus, our primary analysis used generalized estimating equations, but our secondary analysis used mixed models to assess the robustness of the results to differences in statistical assumptions. The results did not differ qualitatively when mixed models were used instead of generalized estimating equations, which implies that our observations were robust.

### Limitations

This analysis has several limitations. We lacked information about some key potential confounders, such as the quality of EMS cardiopulmonary resuscitation^52,53,54^ and the quality of hospital-based postresuscitation care.^55^ This analysis included only data from the US, so it should be replicated in other settings in which the associations among rurality, economic deprivation, and patient outcomes may differ.

We assessed discharge destination rather than described neurologic outcome by electronic medical record abstraction. In prior studies, the CPC^56^ has often been used to assess neurologic status at discharge after OHCA based on manual review of the clinical record. Recently, CPC has been superseded by the modified Rankin score based on the clinical record, as the latter has greater granularity. Since no manual review is performed within our registry, CPC or Rankin score is not routinely collected as part of the registry. Author R.H.S. reported that the CPC at discharge has a moderate correlation with discharge destination (*r*^2^ = 0.63 among 4080 patients enrolled in Resuscitation Outcomes Collaboration Epistry with both elements available) (written communication, May 25, 2024).

Our analyses of outcomes at hospital discharge lacked precision (ie, had a smaller sample size) vs our analyses of ROSC at ED arrival. Traditionally, assessments of the effect of resuscitation interventions have focused on the former rather than the latter.^57,58^ However, early withdrawal of life-sustaining treatment is common among patients hospitalized after resuscitation from cardiac arrest^59^ and confounds assessment of the effectiveness of field interventions.^60^ As well, the wide variation in use of hospital-based hypothermia or targeted temperature management modifies the effect of field interventions.^61^ Thus, we believe that ROSC at ED arrival has greater utility than hospital outcomes for assessments of the effectiveness of field interventions in patients with OHCA. We expect that the difference in precision with analyses of ED vs hospital outcomes using these data may diminish over time as more institutions adopt the use of the health data exchange mechanism in this registry.

Rurality and deprivation were grouped at the EMS agency level rather than by street address where the OHCA occurred, and we acknowledge that this was inexact. However, using the street address is also inexact as it represents where the incident occurred rather than where the patient lives.

Alternative cutoffs for defining urban vs rural with RUCA codes include 4 or higher as rural, a commonly used cutoff in the rural health literature.^62^ We opted to use a different cutoff to give our results additional granularity. Alternative measures of rurality also exist.^63,64^ Studies have shown that the choice of rurality measure is significantly associated with research outcomes.^65,66^ We did not use urban influence codes, another commonly used measure of rurality because they are grouped at the county level, and we were aware that some EMS agencies served areas smaller than this. Post hoc analyses did not suggest that use of alternative cutoffs would alter associations (eFigures 1-6 in Supplement 1).

Alternative measures of neighborhood deprivation exist.^67^ The Social Vulnerability Index includes multiple components, including minority status. Because we did not use this index, the association between patient race or ethnicity and outcome could be incorporated into all models separately. Recently, the ADI has been reported to sometimes yield implausible rankings of community deprivation, which was attributed to lack of standardization of measures prior to calculating index scores.^68^ This debate became public after we developed our statistical analysis plan. We did not modify our plan post hoc to reduce potential overfitting and bias.

We expected a priori that there would be an interaction between rurality and deprivation. The *P* value for this interaction was not significant in some models and not calculable in others due to the sparse numbers in some cells. We kept the interaction in the models as we defined it a priori, the models with an interaction term had good fit, and we were aware that tests for the significance of interaction terms are not very powerful.^69^

## Conclusions

This cohort study found an association between patients with OHCA in rural areas with any degree of economic deprivation and a low likelihood of achieving ROSC at ED arrival. Patients with OHCA in urban areas with moderate or high deprivation had a significantly lower odds of survival to hospital discharge or a favorable discharge destination. Nonclinical and clinical strategies may be necessary to reduce geographic variations in outcomes after OHCA.

## Notes

Supplement 1. **eTable 1.** Comparison of Main and Interaction Effects Between GEE and LMM **eTable 2.** Rurality and Deprivation in Separate Models **eTable 3.** Continuous Rurality and Deprivation Scores vs Outcomes, Adjusted for Covariates **eFigure 1.** Rural Urban Commuting Area vs Restoration of Circulation at Emergency Department Arrival **eFigure 2.** Rural Urban Commuting Area vs Survival to Discharge **eFigure 3.** Rural Urban Commuting Area vs Favorable Neurologic Outcome **eFigure 4.** Area Deprivation Index vs Restoration of Circulation at Emergency Department Arrival **eFigure 5.** Area Deprivation Index vs Survival to Discharge **eFigure 6.** Area Deprivation Index vs Favorable Neurologic Outcome

Supplement 2.
Data Sharing Statement
