# Supplementary material for: Rurality and Area Deprivation and Outcomes After Out-of-Hospital Cardiac Arrest
Source: JAMA Netw Open. 2025 Apr 15;8(4):e253435. doi: 10.1001/jamanetworkopen.2025.3435 (PMC12000968; doi:10.1001/jamanetworkopen.2025.3435)
Supplement: Supplement 1. — eTable 1. Comparison of Main and Interaction Effects Between GEE and LMM eTable 2. Rurality and Deprivation in Separate Models eTable 3. Continuous Rurality and Deprivation Scores vs Outcomes, Adjusted for Covariates eFigure 1. Rural Urban Commuting Area vs Restoration of Circulation at Emergency Department Arrival eFigure 2. Rural Urban Commuting Area vs Survival to Discharge eFigure 3. Rural Urban Commuting Area vs Favorable Neurologic Outcome eFigure 4. Area Deprivation Index vs Restoration of Circulation at Emergency Department Arrival eFigure 5. Area Deprivation Index vs Survival to Discharge eFigure 6. Area Deprivation Index vs Favorable Neurologic Outcome [file jamanetwopen-e253435-s001.pdf]

Supplemental Online Content

Cheek L, Schmicker RH, Crowe R, et al. Rurality and area deprivation and outcomes after out-of-hospital cardiac arrest. *JAMA Netw Open*. 2025;8(4):e253435.
doi:10.1001/jamanetworkopen.2025.3435

eTable 1. Comparison of Main and Interaction Effects Between GEE and LMM
eTable 2. Rurality and Deprivation in Separate Models
eTable 3. Continuous Rurality and Deprivation Scores vs Outcomes, Adjusted for Covariates
eFigure 1. Rural Urban Commuting Area vs Restoration of Circulation at Emergency Department Arrival
eFigure 2. Rural Urban Commuting Area vs Survival to Discharge
eFigure 3. Rural Urban Commuting Area vs Favorable Neurologic Outcome
eFigure 4. Area Deprivation Index vs Restoration of Circulation at Emergency Department Arrival
eFigure 5. Area Deprivation Index vs Survival to Discharge
eFigure 6. Area Deprivation Index vs Favorable Neurologic Outcome

This supplemental material has been provided by the authors to give readers additional information about their work.

© 2025 Cheek L et al. *JAMA Network Open*.

eTable 1. Comparison of Main and Interaction Effects Between GEE and LMM

		OR (95% CI)					
**Outcome**		**ROSC at ED**		**Survival to Discharge**		**Favorable Neurologic Outcome**	
**model**		**GEE**	**LMM**	**GEE**	**LMM**	**GEE**	**LMM**
**Rurality**	**Deprivation**						
Rural	High	0.81 (0.72, 0.91)	0.86 (0.88, 0.98)	0.81 (0.32, 2.05)	1.09 (0.34, 3.17)	0.32 (0.06, 1.69)	0.49 (0.12, 4.11)
Rural	Moderate	0.75 (0.70, 0.81)	0.77 (0.92, 0.84)	0.92 (0.67, 1.28)	1.18 (0.68, 1.74)	0.65 (0.40, 1.07)	0.97 (0.58, 1.67)
Rural	Low	0.74 (0.62, 0.88)	0.77 (0.82, 0.94)	1.14 (0.57, 2.28)	1.29 (0.48, 2.70)	0.41 (0.14, 1.22)	0.61 (0.32, 1.92)
Suburban	High	0.94 (0.86, 1.03)	0.95 (0.90, 1.05)	1.05 (0.69, 1.59)	1.27 (0.64, 1.98)	0.66 (0.35, 1.22)	0.88 (0.53, 1.67)
Suburban	Moderate	0.85 (0.82, 0.89)	0.86 (0.94, 0.92)	0.94 (0.81, 1.09)	0.96 (0.84, 1.14)	0.87 (0.71, 1.06)	1.00 (0.80, 1.26)
Suburban	Low	0.81 (0.75, 0.87)	0.83 (0.92, 0.89)	1.04 (0.83, 1.31)	1.10 (0.79, 1.39)	0.84 (0.61, 1.14)	1.02 (0.72, 1.42)
Urban	High	1.07 (1.01, 1.13)	1.03 (0.94, 1.10)	0.79 (0.69, 0.90)	0.93 (0.85, 1.09)	0.65 (0.53, 0.79)	0.74 (0.80, 0.92)
Urban	Moderate	0.99 (0.95, 1.02)	0.98 (0.96, 1.02)	0.82 (0.75, 0.89)	0.87 (0.90, 0.96)	0.77 (0.69, 0.87)	0.81 (0.87, 0.92)
Urban	Low	reference	reference	reference	reference	reference	reference

© 2025 Cheek L et al. *JAMA Network Open*.

eTable 2. Rurality and Deprivation in Separate Models

Outcome	ROSC at ED	Survival to Discharge	Favorable Neurologic Outcome
n	128641	18125	17347
**RUCA Classification**			
**Urban**	reference	reference	reference
**Suburban**	0.85 (0.82, 0.88)	1.10 (0.97, 1.24)	0.97 (0.82, 1.15)
**Rural**	0.76 (0.72, 0.81)	1.07 (0.80, 1.42)	0.65 (0.42, 1.01)
Age>=65	0.83 (0.81, 0.86)	0.64 (0.60, 0.69)	0.41 (0.37, 0.46)
Gender			
Female	reference	reference	reference
Male	0.76 (0.74, 0.79)	0.88 (0.81, 0.95)	1.00 (0.89, 1.11)
Location			
Public	1.29 (1.24, 1.35)	1.36 (1.24, 1.50)	1.62 (1.43, 1.83)
Private	reference	reference	reference
Initial Rhythm			
VTVF	reference	reference	reference
Asystole	0.40 (0.39, 0.42)	0.22 (0.20, 0.24)	0.15 (0.13, 0.18)
PEA	0.87 (0.84, 0.91)	0.46 (0.42, 0.51)	0.35 (0.31, 0.39)
Arrival Time (min)	0.99 (0.99, 0.99)	0.97 (0.96, 0.99)	0.99 (0.96, 1.01)
Race			
Asian, non-Hispanic	1.18 (1.07, 1.30)	1.18 (0.92, 1.52)	1.07 (0.76, 1.51)
Black, non-Hispanic	0.86 (0.83, 0.89)	0.91 (0.83, 1.00)	0.84 (0.74, 0.96)
Hispanic	0.95 (0.90, 1.00)	1.10 (0.97, 1.26)	1.09 (0.91, 1.31)
White	reference	reference	reference
Other race, non-Hispanic	0.80 (0.67, 0.95)	0.89 (0.58, 1.36)	0.89 (0.47, 1.67)
Bystander CPR	1.05 (1.02, 1.09)	1.10 (1.01, 1.20)	1.20 (1.06, 1.36)
Witness Status			
Not	reference	reference	reference
Bystander	2.02 (1.95, 2.08)	1.66 (1.50, 1.82)	1.98 (1.71, 2.29)
EMS	2.39 (2.29, 2.50)	1.98 (1.77, 2.23)	2.67 (2.26, 3.15)
Transport Time (min)	--	1.01 (1.01, 1.02)	1.02 (1.02, 1.03)

	OR (95% CI)		
Outcome	ROSC at ED	Survival to Discharge	Favorable Neurologic Outcome
n	128641	18125	17347
**Deprivation**			
**Low**	reference	reference	reference
**Moderate**	0.96 (0.93, 0.98)	0.84 (0.77, 0.91)	0.80 (0.72, 0.90)
**High (**	1.03 (0.99, 1.08)	0.80 (0.71, 0.91)	0.65 (0.54, 0.79)
Age>=65	0.83 (0.81, 0.86)	0.63 (0.59, 0.68)	0.40 (0.36, 0.45)
Gender			
Female	reference	reference	reference
Male	0.76 (0.74, 0.79)	0.87 (0.80, 0.94)	0.99 (0.89, 1.11)
Location			
Public	1.30 (1.25, 1.36)	1.35 (1.23, 1.49)	1.59 (1.41, 1.80)
Private	reference	reference	reference
Initial Rhythm			
VTVF	reference	reference	reference
Asystole	0.40 (0.39, 0.42)	0.22 (0.20, 0.24)	0.15 (0.13, 0.18)
PEA	0.88 (0.84, 0.91)	0.46 (0.42, 0.51)	0.35 (0.31, 0.39)
Arrival Time (min)	0.99 (0.98, 0.99)	0.97 (0.96, 0.99)	0.98 (0.96, 1.01)
Race			
Asian, non-Hispanic	1.23 (1.11, 1,35)	1.11 (0.86, 1.42)	1.01 (0.72, 1.42)
Black, non-Hispanic	0.88 (0.85, 0.91)	0.93 (0.85, 1.03)	0.89 (0.78, 1.01)
Hispanic	0.97 (0.92, 1.02)	1.11 (0.86, 1.42)	1.01 (0.72, 1.42)
White	reference	1.08 (0.94, 1.24)	1.07 (0.89, 1.28)
Other race, non- Hispanic	0.80 (0.67, 0.96)	0.85 (0.56, 1.30)	0.86 (0.46, 1.61)
Bystander CPR	1.04 (1.01, 1.08)	1.09 (1.00, 1.19)	1.19 (1.05, 1.34)
Witness Status			
Not	reference	reference	reference
Bystander	2.00 (1.93, 2.06)	1.66 (1.51, 1.82)	1.97 (1.70, 2.27)
EMS	2.37 (2.27, 2.47)	1.99 (1.78, 2.23)	2.67 (2.26, 3.16)
Transport Time (min)	--	1.01 (1.01, 1.02)	1.02 (1.01, 1.03)

© 2025 Cheek L et al. *JAMA Network Open*.

eTable 3. Continuous Rurality and Deprivation Scores vs Outcomes, Adjusted for Covariates

	OR (95% CI)		
**Outcome**	**ROSC at ED**	**Survival to Discharge**	**Favorable Neurologic Outcome**
n	128641	18125	17347
C statistic	0.69	0.72	0.78
Rurality (RUCA Score, unit of 1)	0.94 (0.91, 0.96)	1.02 (0.92, 1.13)	0.87 (0.76, 1.01)
Deprivation (ADI Score, unit of 10)	1.00 (0.99, 1.01)	0.96 (0.93, 0.98)	0.93 (0.90, 0.96)
Age>=65	0.84 (0.81, 0.86)	0.63 (0.58, 0.68)	0.40 (0.36, 0.45)
Gender			
Female	reference	reference	reference
Male	0.77 (0.74, 0.79)	0.87 (0.81, 0.94)	0.99 (0.89, 1.11)
Race			
Asian, non-Hispanic	1.21 (1.10, 1.33)	1.10 (0.85, 1.41)	0.98 (0.69, 1.38)
Black, non-Hispanic	0.86 (0.83, 0.89)	0.95 (0.86, 1.04)	0.88 (0.77, 1.01)
Hispanic	0.96 (0.91, 1.01)	1.08 (0.95, 1.24)	1.07 (0.89, 1.28)
White, non-Hispanic	reference	reference	reference
Other race, non-Hispanic	0.81 (0.67, 0.97)	0.86 (0.56, 1.31)	0.85 (0.45, 1.60)
Location			
Public	1.30 (1.25, 1.36)	1.35 (1.22, 1.48)	1.59 (1.41, 1.80)
Private	reference	reference	reference
Bystander CPR	1.05 (1.02, 1.09)	1.09 (1.00, 1.19)	1.19 (1.05, 1.34)
Witness Status			
Not	reference	reference	reference
Bystander	2.01 (1.95, 2.08)	1.65 (1.50, 1.82)	1.97 (1.70, 2.27)
EMS	2.38 (2.28, 2.48)	1.99 (1.77, 2.23)	2.67 (2.26, 3.16)
Initial Rhythm			
VTVF	reference	reference	reference
Asystole	0.40 (0.39, 0.41)	0.22 (0.20, 0.24)	0.15 (0.13, 0.18)
PEA	0.87 (0.84, 0.91)	0.47 (0.42, 0.51)	0.35 (0.31, 0.39)
Arrival Time (min)	0.99 (0.99, 0.99)	0.97 (0.96, 0.99)	0.98 (0.96, 1.01)
Transport Time (min)	--	1.00 (0.99, 1.02)	1.02 (1.00, 1.04)
Rurality * Deprivation	1.00 (1.00, 1.01)	1.01 (1.00, 1.02)	1.02 (1.01, 1.03)

© 2025 Cheek L et al. *JAMA Network Open*.

eTable 3 (Continued)

The rurality and deprivation variables are each modelled as continuous variable such that rurality estimate is the change in odds of the outcome for one unit difference in rurality where urban = 1 and rural = 10. The deprivation estimate is the change in the odds of the outcome for a 10 unit difference in deprivation. The interaction term for rurality X deprivation represents a one-unit change in the interaction between the two variables.

© 2025 Cheek L et al. *JAMA Network Open*.

eFigure 1. Rural Urban Commuting Area vs Restoration of Circulation at Emergency Department Arrival

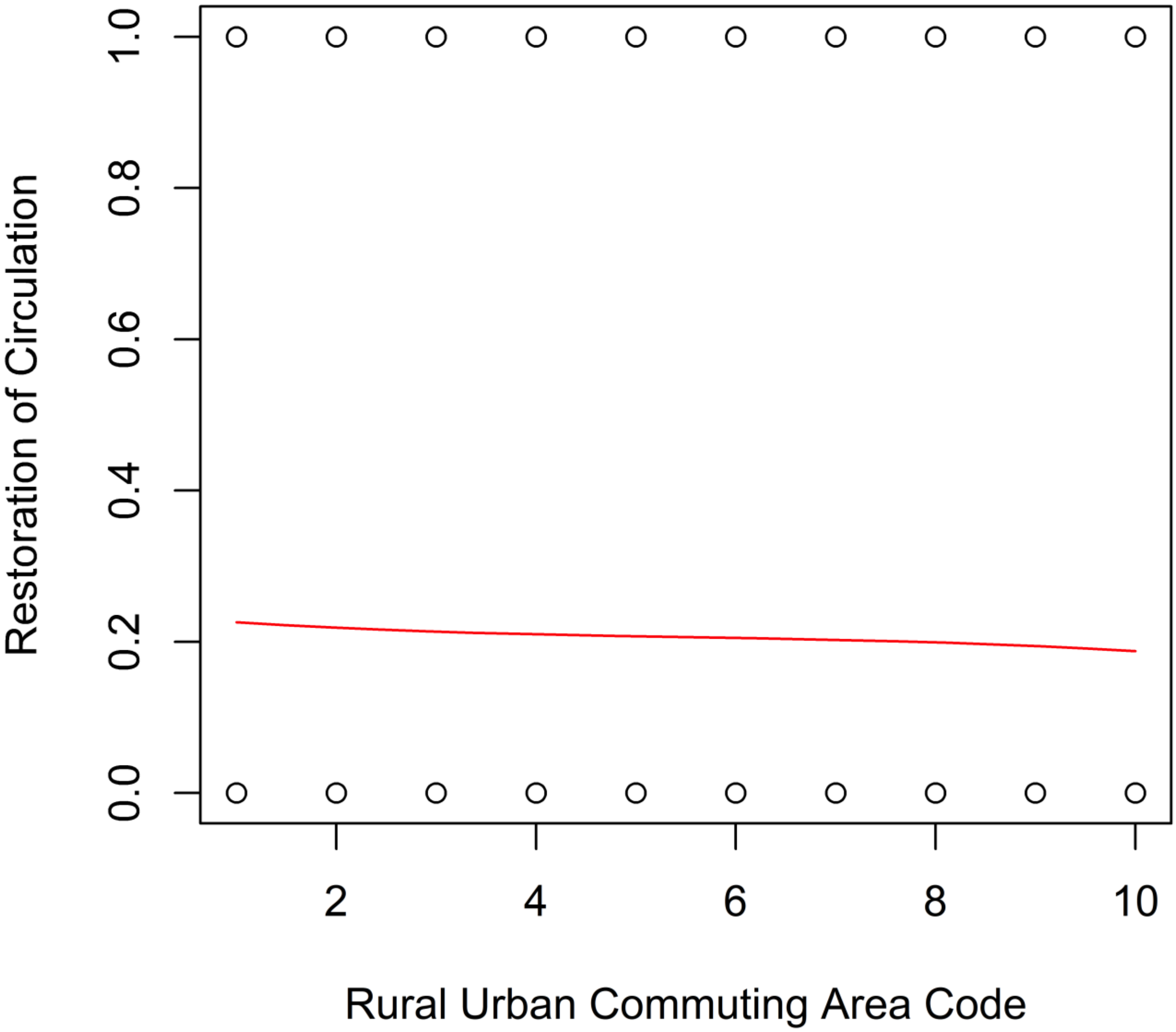

© 2025 Cheek L et al. *JAMA Network Open*.

eFigure 2. Rural Urban Commuting Area vs Survival to Discharge

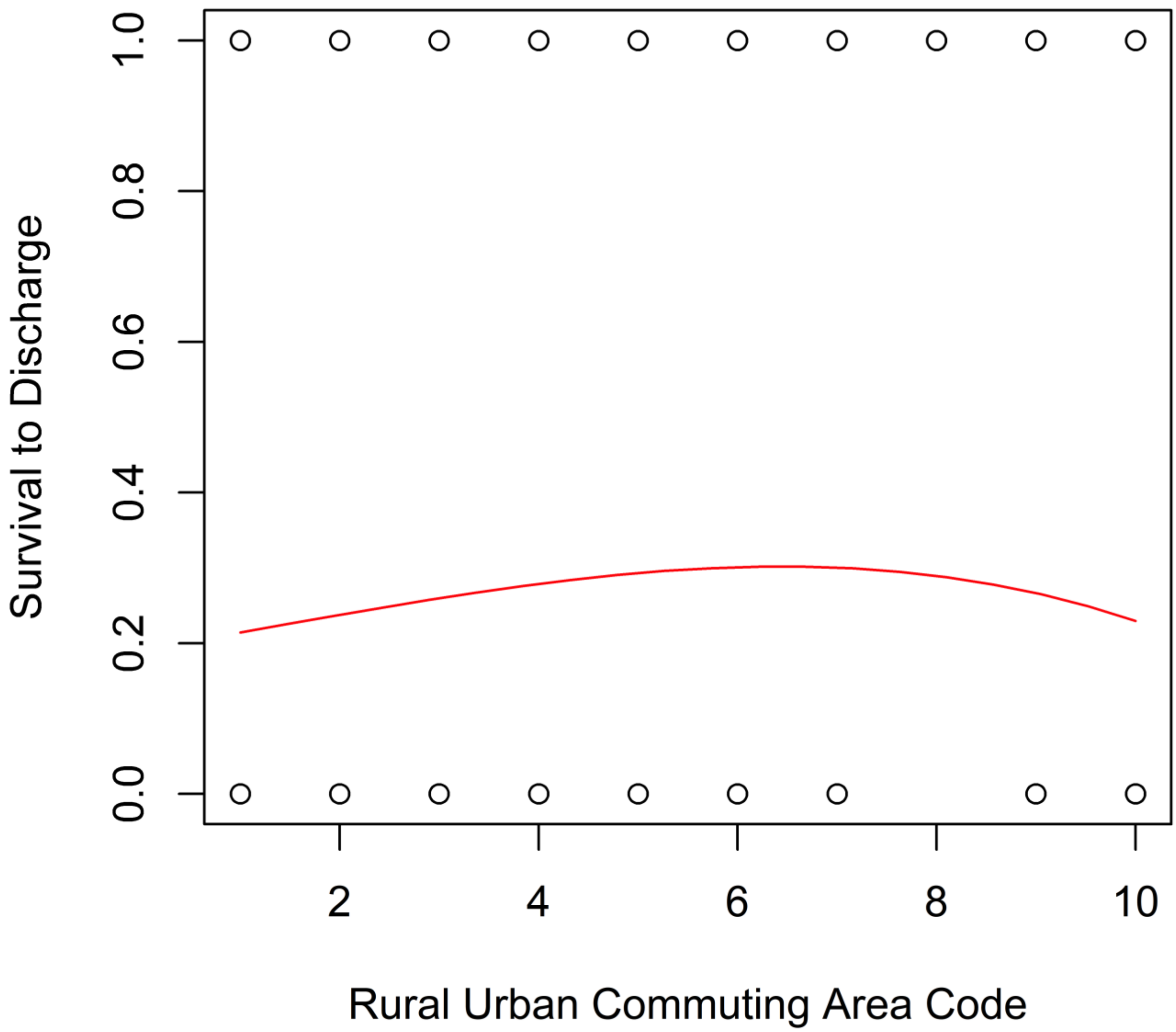

© 2025 Cheek L et al. *JAMA Network Open*.

eFigure 3. Rural Urban Commuting Area vs Favorable Neurologic Outcome

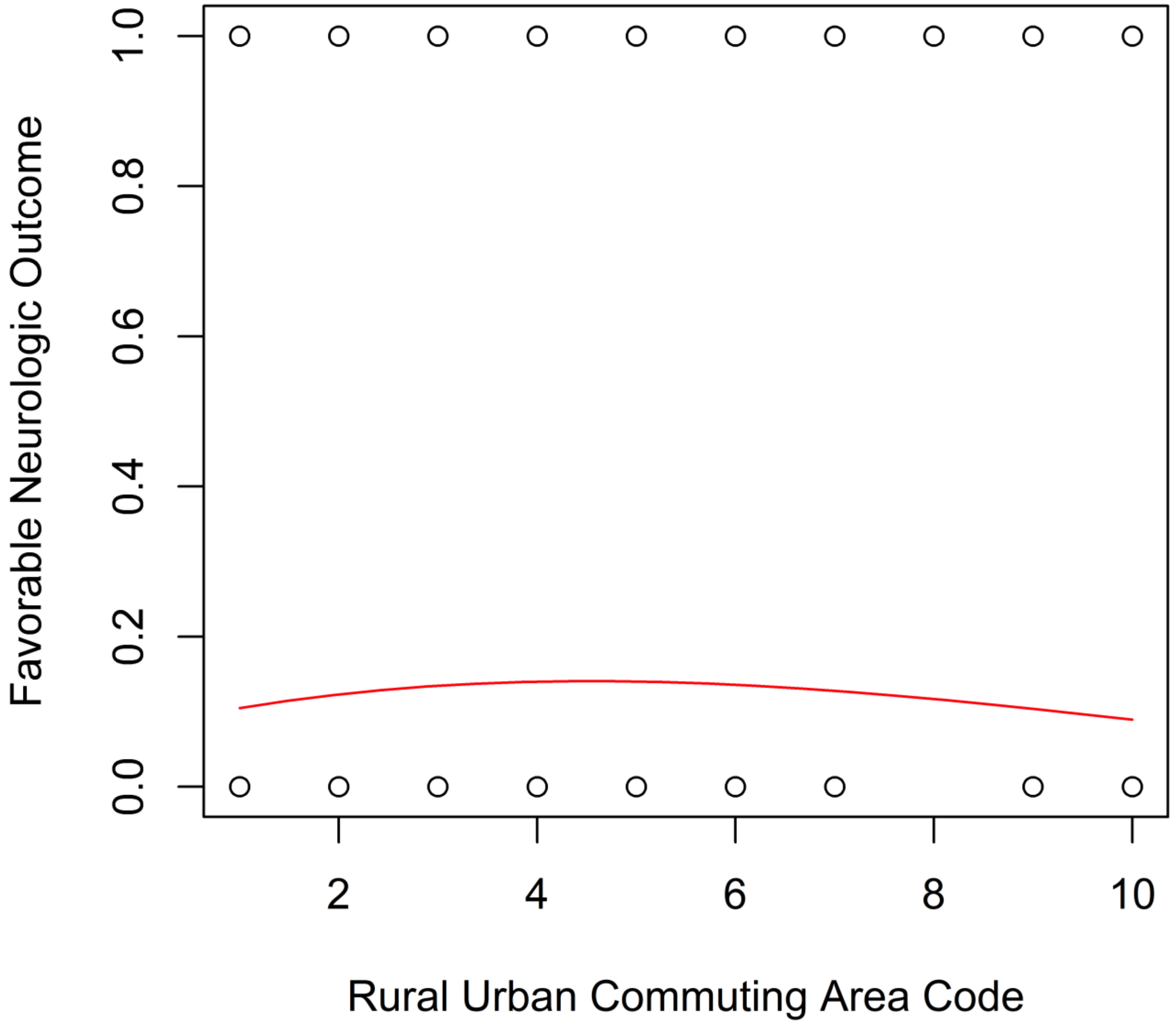

© 2025 Cheek L et al. *JAMA Network Open*.

eFigure 4. Area Deprivation Index vs Restoration of Circulation at Emergency Department Arrival

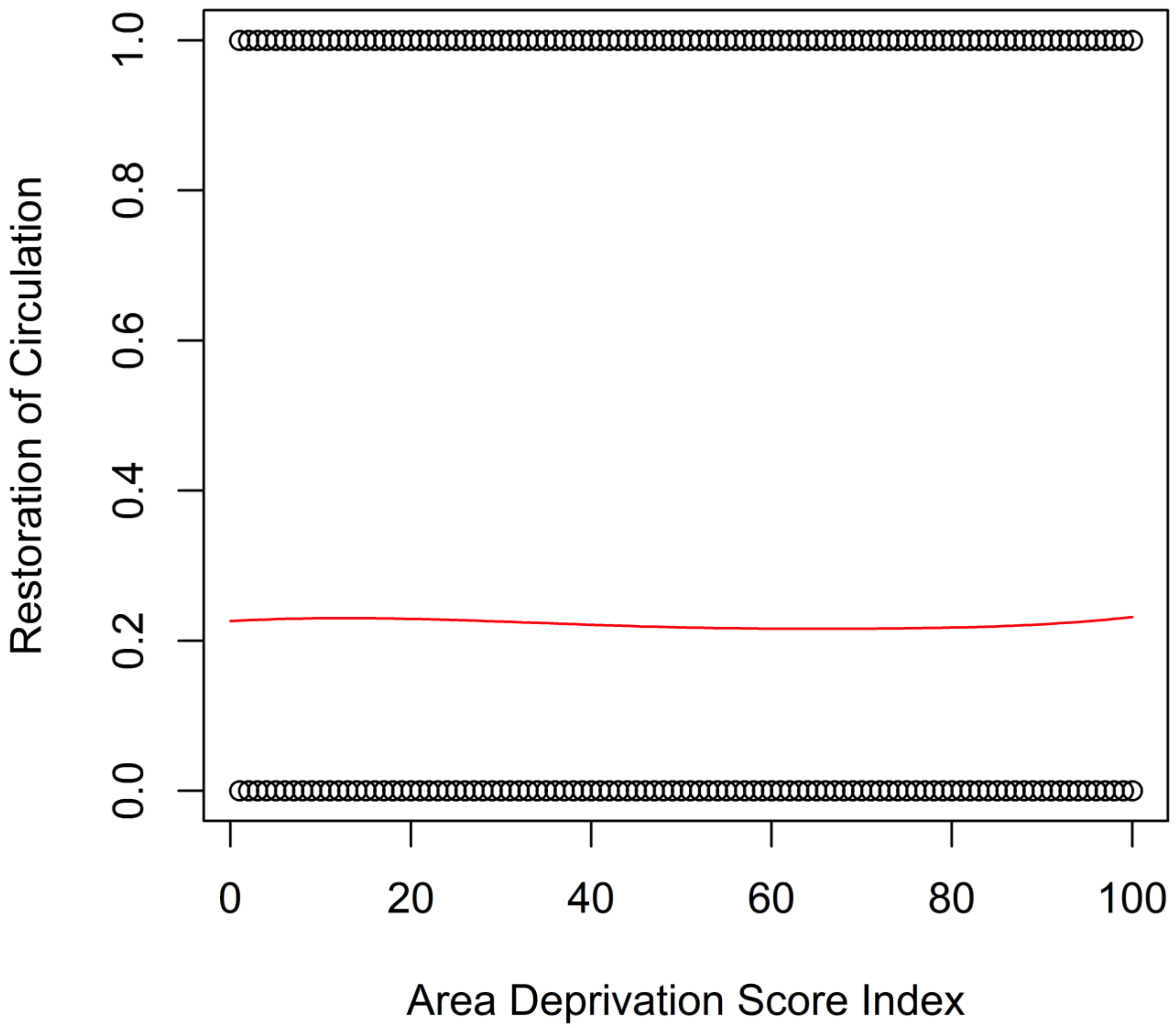

© 2025 Cheek L et al. *JAMA Network Open*.

eFigure 5. Area Deprivation Index vs Survival to Discharge

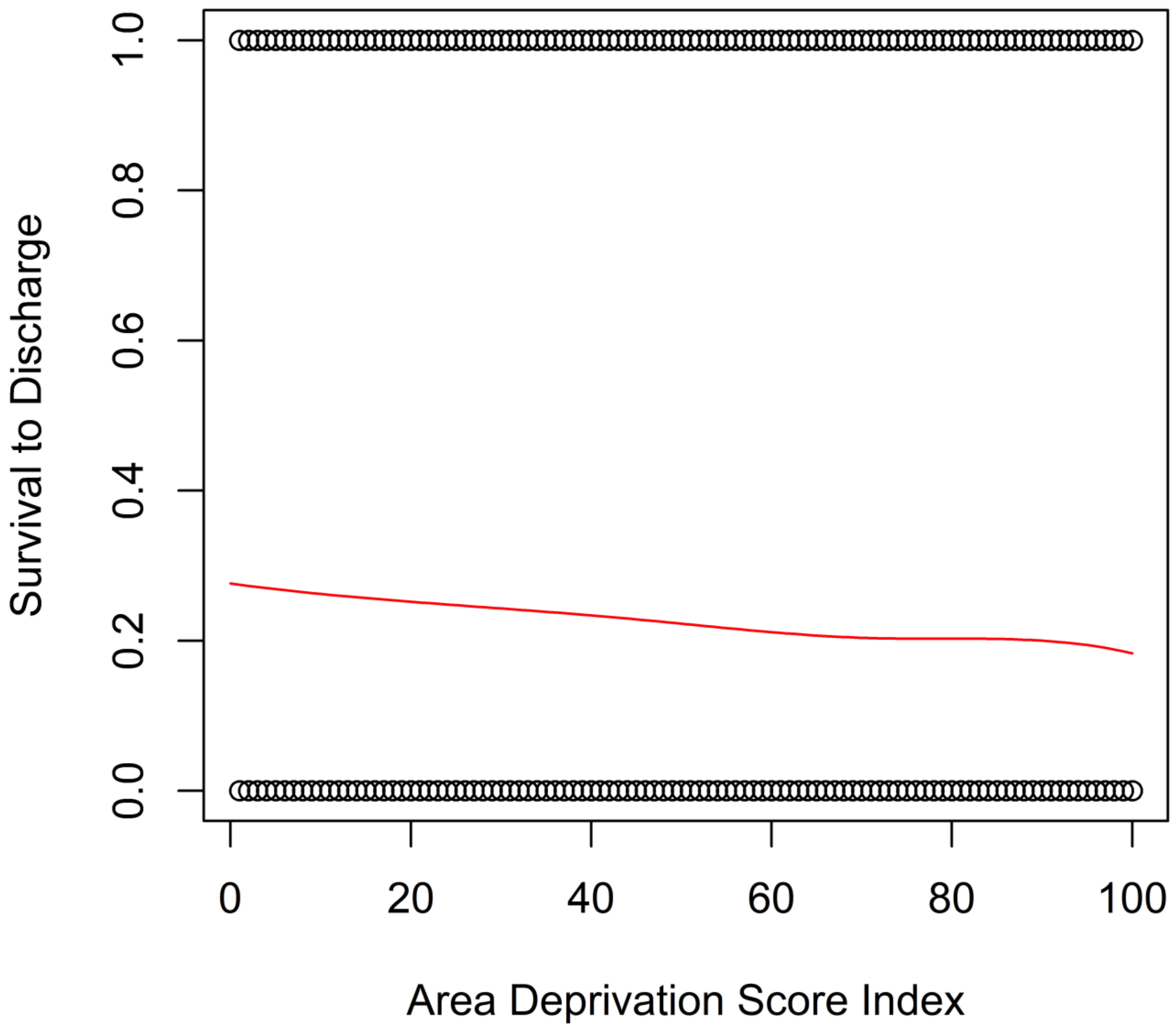

© 2025 Cheek L et al. *JAMA Network Open*.

eFigure 6. Area Deprivation Index vs Favorable Neurologic Outcome

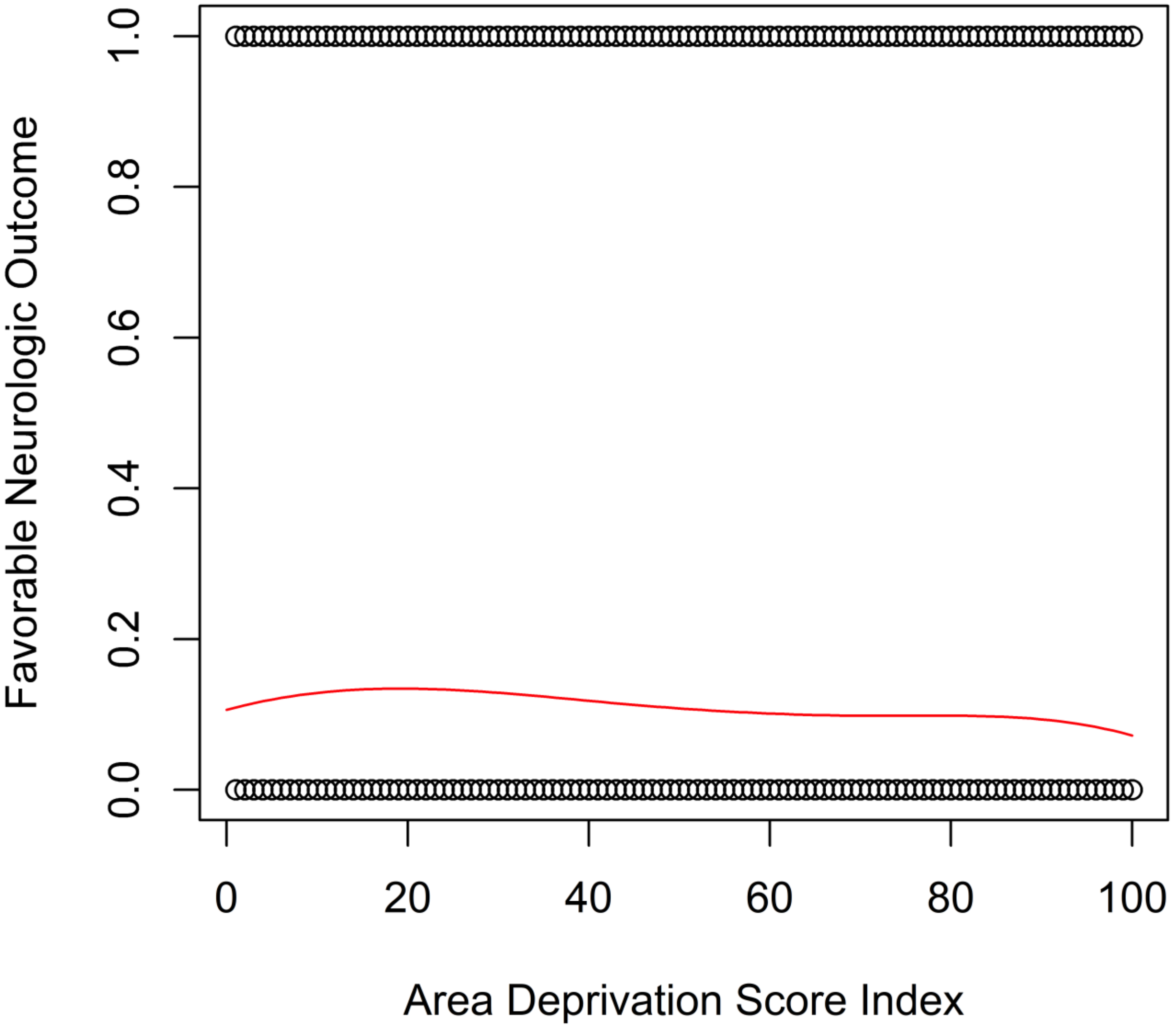

© 2025 Cheek L et al. *JAMA Network Open*.
