# Supplementary material for: Rurality and Area Deprivation and Outcomes After Out-of-Hospital Cardiac Arrest
Source: JAMA Netw Open. 2025 Apr 15;8(4):e253435. doi: 10.1001/jamanetworkopen.2025.3435 (PMC12000968; doi:10.1001/jamanetworkopen.2025.3435)
Supplement: Supplement 2. — Data Sharing Statement [file jamanetwopen-e253435-s002.pdf]

Data Sharing Statement

Cheek. Rurality and Area Deprivation and Outcomes After Out-of-Hospital Cardiac Arrest.
JAMA Netw Open. Published April 15, 2025. doi:10.1001/jamanetworkopen.2025.3435

Data
Data available: No
